# eHealth literacy measurement tools: a systematic review protocol

**DOI:** 10.1186/s13643-022-02076-2

**Published:** 2022-09-24

**Authors:** Carole Délétroz, Marina Canepa Allen, Maxime Sasseville, Alexandra Rouquette, Patrick Bodenmann, Marie-Pierre Gagnon

**Affiliations:** 1 Faculty of Nursing Sciences, Laval University, 2325 Rue de l’Université, Québec, QC G1V 0A6 Canada; 2 School of Health Sciences (HESAV), University of Applied Sciences and Arts Western Switzerland, Avenue de Beaumont 21, 1011 Lausanne, Switzerland; 3 Paris-Saclay University, Inserm, UVSQ, CESP, DevPsy, Paris, France; 4 Public Health and Epidemiology Department, AP-HP Paris-Sarclay, Bicêtre Hospital, Le Kremlin-Bicêtre, France; 5 Department of Vulnerabilities and Social Medicine, Unisanté, Lausanne, Switzerland; 6 Faculty of Biology and Medicine, Vice-Dean Teaching and Diversity, University of Lausanne, Rue du Bugnon 44, 1011 Lausanne, Switzerland

**Keywords:** eHealth literacy, Health literacy, Psychometrics, Systematic review

## Abstract

**Background:**

Improving eHealth literacy (eHL) is one of the biggest challenges currently facing the global healthcare community. Indeed the use of digital services has the potential to engage patients in care as well as improve the effectiveness of chronic disease self-management, it remains highly dependent on a patient’s specific skills and experiences in the health care systems. Although eHealth literacy has gained momentum in the past decade, it remains an underresearched area, particularly eHealth literacy measurement. The aim of the review is to identify patient-reported outcome measures (PROMs) of eHealth literacy for adult populations and to summarize the evidence on their psychometric properties.

**Methods:**

We will conduct a systematic literature review of the tools used to measure eHealth literacy for adult population. The search strategy aims to find published studies. A three-step search strategy will be used in this review. Published studies will be searched in CINAHL, PubMed, PsycINFO, and Web of Science from inception until end. Grey literature will be searched to find theses. Database search strategies will be formulated and tested with the assistance of an expert Health Sciences Librarian. The selection of studies will be done by two independent reviewers. Disagreements will be resolved through consensus, and a third reviewer will solve discrepancies. Furthermore, two reviewers will independently evaluate the methodological rigor of the instruments development and testing and assign a grade using the standardized Consensus-based Standards for the Selection of Health Measurement Instruments (COSMIN) checklist. Disagreements will be discussed with a third reviewer, expert in psychometrics.

Extracted data will be aggregated and analyzed to produce a set of synthesized findings that will be used to develop evidence-informed recommendations in regard of eHL instruments. We will present a synthesis of all instruments, their psychometric properties, and make recommendations for eHL instrument selection in practice. Reporting will be informed by the Preferred Reporting Items for Systematic Reviews and Meta-Analysis and a PRISMA flow diagram.

**Discussion:**

This systematic review will summarize the evidence on the psychometric properties of PROMs instruments used to measure eHL and will help clinicians, managers, and policy-makers to select an appropriate instrument.

**Systematic review registration:**

PROSPERO CRD42021232765

## Background

The generalization of eHealth (electronic health) to numerous activities in the health system is leading to changes in individual and collective health uses and practices. Improving eHealth literacy (eHL), which is a type of health literacy, continues to be a challenge worldwide [1, 2].

A growing number of articles in the literature point out the potential benefits of eHealth interventions to improve health behaviors [3], clinical outcomes [4], and patient empowerment in the management of chronic health conditions [5–7]. Therefore, it is essential for clinicians to evaluate eHL among their patients in order to provide care and services that suit their actual needs and capabilities. eHL was found to be associated with more effective contact with physician, enhance use of medical insurance, improve self-management of health needs, and raise perceived understanding of a disease/condition [8]. Current researchers on eHL emphasize the need to use specific measurement to determine whether eHealth initiatives are improving patient care [5, 9, 10].

For patients, it is becoming critical to have access and skills to understand information through electronic services including health portals or electronic patient record. eHealth referred to “the organization and delivery of health services and information using the Internet and related technologies” [11]. As a result, it requires specific skills to use and interact in the digital environment [8]. Norman and Skinner, taken up by the WHO and the European Commission, described eHL as “the ability to seek, find, understand, and appraise health information from electronic sources and apply the knowledge gained to addressing or solving health problem” ( [1], p. 3). The effectiveness of eHealth is highly dependent on patient eHL’s skills, complementary to health literacy, that are sometimes new to many patients and unevenly distributed across the population. Populations at risk for limited health literacy are similarly vulnerable to eHL challenges, such as the elderly, those with lower education and patients suffering from chronic conditions [12]. Therefore, a certain part of the population will not be able to effectively make use of eHealth services, which could affect health outcomes and the ability to make an optimal choice of health information [13, 14].

## Rationale

eHL is a multidimensional concept with varying definitions [15, 16]. This paper uses the definition of Norman and Skinner, who state that research, evaluation, and patient use of online health information are the three main domains of eHL. eHL is assessed most often by the self-report measure eHEALS [17], which focuses on finding information on the Internet and assessing it. Norman and Skinner [17] found that the measure consists of one factor in an exploratory factor analysis, but recent work [18] uncovered 2 factors: seeking and appraising. Despite the increasing use of the eHEALS, limited evidence exists regarding whether increasing patient’s eHL can improve health outcomes [19]. Measurement of eHL is difficult, because no single measure can capture the different constructs of outcomes of eHealth interventions [9, 20]. Furthermore, eHL should consider not only individual knowledge and skills but also people’s perception of the use of system providing health care as relevant to the management of their condition [21]. Therefore, attempts to assessing eHL by self-reported measure remain challenging in nursing and health research [22, 23].

Several sources of difficulty in measuring eHL can be identified: (1) there is confusion over the term “eHealth literacy” behind the measure [24]. (2) What constitutes eHL within existing instruments is not always defined from the patient’s perspective (validity) [25]. (3) Instruments need to produce consistent and reproducible results (reliability) and, most importantly, instruments need to be suitable for clinical practice [26].

The challenge is to measure eHL as a set of cognitive skills necessary for the effective “use” of eHealth interventions. The literature describes eHL as a metaliteracy that comprises six different subtypes of literacies focusing on the use of online information [15]. The relevance of eHL is demonstrated in recent studies, showing that people’s self-perceived skills to use online information affect health behaviors and self-management of health conditions, and that a lack of such skills may lead to adverse outcomes. Neter and Brainin [8, 27] found relationships between eHL and the presence of chronic illness, perceived self-management skills, and better self-perceived understanding of health status, symptoms, and optional treatments. Hsu and coll [28]. found that eHL skills are associated with various types of positive health behavior, including healthy eating, exercise, and sleep behavior. Furthermore, studies have shown that certain groups do not have the skills and knowledge to use eHealth tools efficiently for their own benefit and might thereby even experiencing difficulty in accessing health care services, such as elderly people, individuals with low health literacy, or presenting with cognitive problems, and those with limited access to technology for various reasons (e.g., limited capacity to use it, inaccessibility to the Internet, chronic degenerative diseases) [29–31]. Due to the possible personal, social, and health impact of eHL, we need to distinguish between instruments measuring patient experiences focusing only on the use of online information and those considering skills needed in using eHealth.

A self-reported measure of a patient’s eHL needs to be based on sound theoretical and empirical evidence and those instruments should be representative of patients’ experience of eHL in daily life (content validity). To date, there is only one self-report measure used in more than one study: the eHealth Literacy Scale (eHEALS) [17]. This measure focuses on finding information on the Internet and assessing it. A broader self-report scale that also addresses generating information was just recently developed [32]; however, only few eHL’s studies were performed [10, 33]. There is a risk that self-reported instruments have been developed based on literature only, or from experts’ opinions about important aspects of eHL, without considering patients experiencing eHealth in their personal background. A valid measurement instrument of eHL is essential to examine the effects of eHealth, both on an individual level and on a population level. On an individual level—for example, in daily clinical practice—a measurement instrument could support decisions about the extent to which a patient is able to benefit from particular eHealth tools and interventions. On a population level, a proper measurement instrument could provide insight into vulnerable subgroups that face additional challenges in using health care, due to its digitization. It is imperative to determine the validity of existing eHL’s self-reported measure. Besides, instruments measuring the patients’ eHL must be acceptable and interpretable to both patients and clinicians.

The standardized Consensus-based Standards for the selection of health Measurement Instruments (COSMIN) risk of bias checklists [34, 35] provides a framework for the quality assessment of each included study and the determination of a grade of evidence of health measurement instruments. The quality assessment of each included study is assessed using the “scoring quality form COSMIN boxes” [36]. The overall rating (grade evidence) is based on the quality criteria for good measurement properties proposed by the COSMIN group [37].

The measures of eHL need to produce consistent and reproducible results if they are to be trusted in practice (reliability). These measures are PROMs, which are defined as standardized, validated questionnaires (which are also called instruments) completed by patients [36]. Data arising from PROMs may be used to identify skills for adequate use of eHealth tools and interventions [38]. Therefore, the psychometrics of each measure of eHL need to be evaluated and described to facilitate their use in the relevant context.

Currently, there is one systematic review [19] that identified PROMs of eHL and describes the conceptual models they are based on. The authors identified eight instruments, with only three of them being based upon a conceptual model of eHealth literacy: eHEALS, eHLS, and PRE-HIT. The remaining five instruments were dual tools, i.e., comprised of individual measures for health literacy and E-literacy [19]. This review mentions lacks assessment of the quality (i.e., risk of bias) of published study on reliability or measurement error of each instrument. There is a necessity to consider theoretical and empirical issues to be able to select the right instrument for the right purpose.

The current systematic review seeks to examine the quality of PROMs (used for patients: adults > 18 years old) and to identify the best instrument for field use. These findings could assist in informing evidence-based practice for the use of PROMs of eHL. We expect that the findings will assist researchers and clinicians in identifying and selecting the most appropriate instrument when measuring eHL.

## Study aim and research question

The aim of this review is to systematically identify, synthesize, and evaluate the methodological quality of all relevant studies on the development and validation of eHL PROMs for adult populations. The research question for this review is: which PROMs have been developed to assess eHL and what are their psychometric qualities?

## Method

### Study method

Following the methods for conducting systematic reviews outlined in the Cochrane Handbook [39], we developed a review protocol in PROSPERO (registration number CRD42021232765). The Preferred Reporting Items For Systematic Reviews and Meta-Analysis (PRISMA-P) [40] and a PRISMA flow diagram [41] will be used to ensure the design and reporting quality of the review. The evaluation of the methodological rigor of the testing of the instruments and determination of the Grade will be done with the standardized Consensus-based Standards for the Selection of Health Measurement Instruments (COSMIN) checklist [36].

### Search strategy

The search strategy will aim to find published studies describing the development and the validation of eHL self-reported instrument from their inception until July 2021. A three-step search strategy will be used in this review [39]. An initial limited search of PubMed and CINAHL has been undertaken with a screening of the words contained in the title and abstract, looking for terms, keywords, and index term used in relevant articles, and of the index terms used to describe each article. This informed the development of a search strategy which will be tailored for each information source. A second search will be done across four databases: CINAHL, PubMed, PsycINFO, and Web of Science. Database search strategies will be formulated and tested with the assistance of an expert Health Sciences Librarian from Laval University (Laferrière M-C). ProQuest dissertations and Thesis, Opengrey, DART, and BASE will be consulted in order to find theses in the grey literature. Thirdly, the reference list of all identified reports and articles will be screened for additional studies. A sample search strategy from MEDLINE is presented below (Table 1).

**Table 1:** Search strategy PubMed

#	Question	Results
16	((#6 AND #9) OR #3) AND (#13 OR #14)	1,912
15	#13 OR #14	6,048,301
14	"Reproducibility of Results"[Mesh] OR "Psychometrics"[Mesh] OR "Surveys and Questionnaires"[Mesh] OR "Interviews as Topic"[Mesh]	1,444,624
13	Psychometr*[Title/Abstract] OR validity[Title/Abstract] OR measure*[Title/Abstract] OR "quantitative evaluation*"[Title/Abstract] OR scale*[Title/Abstract] OR scales[Title/Abstract] OR instrument*[Title/Abstract] OR tool[Title/Abstract] OR tools[Title/Abstract] OR toolkit*[Title/Abstract] OR questionnaire*[Title/Abstract] OR survey*[Title/Abstract] OR interview*[Title/Abstract]	5,456,755
12	(#6 AND #9) OR #3	2,736
11	#6 AND #9	2,493
9	#8 OR #7	2,361,309
8	"Internet"[Mesh] OR "Digital Divide"[Mesh] OR "Computers"[Mesh] OR "Computer Literacy"[Mesh] OR "Telemedicine"[Mesh:NoExp] OR "Mobile Applications"[Mesh] OR "Online Social Networking"[Mesh] OR "Patient Portals"[Mesh]	177,289
7	digital[Title/Abstract] OR computer*[Title/Abstract] OR informatic*[Title/Abstract] OR electronic*[Title/Abstract] OR internet[Title/Abstract] OR app[Title/Abstract] OR apps[Title/Abstract] OR application*[Title/Abstract] OR mobile[Title/Abstract] OR Telehealth[Title/Abstract] OR telemedicine[Title/Abstract] OR technolog*[Title/Abstract] OR "social media"[Title/Abstract] OR "social computing"[Title/Abstract] OR "social network*"[Title/Abstract] OR "patient portal"[Title/Abstract] OR smartphone [Title/Abstract]	2,287,365
6	(("health literac*"[Title/Abstract])) OR ("health literacy"[MeSH Terms])	10,604
5	"health literacy"[MeSH Terms]	5,79
4	("health literac*"[Title/Abstract])	8,546
3	(eHealth[Title/Abstract] OR e-Health[Title/Abstract] OR mhealth[Title/Abstract] OR m-health[Title/Abstract]) AND (literac*[Title/Abstract])	608
2	literac*[Title/Abstract]	19,618
1	eHealth[Title/Abstract] OR e-Health[Title/Abstract] OR mhealth[Title/Abstract] OR m-health[Title/Abstract]	11,076

Finally, a last search on PubMed with a filter specifically created for the search of PROMs will be done [42] and MeSh will be updated, if necessary, by M-C L. For each measure of eHL identified, we will do a specific search and if needed, we will contact the authors to identify any relevant studies. Following the search, all identified citations will be collated and uploaded into EndNote X9 [43]. Duplicates will be removed manually.

### Inclusion criteria

This review will consider all studies conducted on the development and/or validation (including psychometric quality) of measures of eHL in adults (> 18 years old). Studies relating to human health and published between 2000 and 2021 in English, French, Spanish, or Italian will be considered for inclusion. The adoption of e-health by health institutions start in the early 2000’s with the rapid emergence of technology use during that time [44]. Therefore, only studies published from 2000 onwards will be considered for inclusion in this review. eHL of children and teenagers is conceptualized in a contradictory way in the context of digital health skills: firstly, the “digital natives” are imagined as particularly competent and user of the digital world, secondly, they are seen as a particular risk group in the context of internet addiction, thirdly, empirical studies show that adolescents in particular consider themselves to be more competent in health than adults in self-reporting [45]. Therefore, studies measuring eHL among children (< 18 years old) will not be included. Table 2 presents the outcomes of the review according to COSMIN’s methodology.

**Table 2:** Outcomes–measurement quality criteria according to Terwee

1	*Internal consistency: Cronbach’s alpha (Cronbach (*α*)), Kunder-Richardson’s coefficient (KR20 et KR21), *χ*2
2	*Reliability, relative measures (including test-retest reliability, inter-rater reliability and intra- rater reliability): intraclass correlation coefficient (ICC), Kappa coefficient (*K*), the weighting scheme description—e.g., linear quadratic
3	*Measurement error, absolute measures: standard error of measurement (SEM), smallest detectable change (SDC), or limits of agreement (LoA)
4	*Content validity, including face validity: qualitative outcomes about concept elicitation, relevance and comprehensiveness by asking patients and/or professionals, and about comprehensibility and comprehensiveness of the PROMs.
5	*Construct validity: Structural validity: results of exploratory (EFA) or confirmatory factor analysis (CFA); results of item response theory (IRT) test for determining the (uni-) dimensionality of the scale**.** Hypotheses testing, design and statistical methods adequate for the hypotheses being tested Cross-cultural validity: confirmatory factor analysis (CFA); differential item function (DIF) between language groups assessed
6	*Responsiveness (no gold standard DHL measure available): design and statistical methods adequate for the hypotheses to be tested

### Study selection (selection process)

Titles and abstracts will be screened against the inclusion criteria for the review. Studies that meet the inclusion criteria will be retrieved in full (including full text) and their citation details imported into Covidence [46]. The full text of selected citations will be assessed in detail against the inclusion criteria by two independent reviewers. Reasons for exclusion of full-text studies will be recorded and reported in the final systematic review. Any disagreements that arise between the reviewers at each stage of the study selection process will be resolved through discussion with a third reviewer. The results of the search will be reported in full in the final report and presented in a Preferred Reporting Items for Systematic Reviews and Meta-analyses (PRISMA) flow diagram [40, 47].

### Patient and public involvement

This research will be done without patient involvement. As this review aims at synthesizing methodological information about existing eHL instruments, patient and public experience is not directly mobilized. However, patient and public will be involved at a later stage in this project.

### Data extraction

Data will be extracted from the selected studies using the data extraction form (Table 3) proposed by the Consensus-based Standards for the Selection of Health Measurement Instruments (COSMIN) and the Risk of bias checklist [34] that will be integrated in Covidence.

**Table 3:** Data extraction form

General information	Author Year Country of origin Papers
Instrument details	eHL instrument name HL domain and component (item number) Target population Response category Scoring system (range of score) Burden (minutes for administration) Original language
Characteristics of PROM	PROM development Content validity Structural validity Internal consistency Cross-cultural validity\Measurement invariance Reliability Measurement error Criterion validity Hypotheses testing for construct validity Responsiveness

Two researchers will independently extract the data for all included studies and agree, through consensus, about the accuracy and completeness of the data. Where consensus is difficult to achieve, a third reviewer will be used to reach agreement verification (including psychometrics) to minimize bias and potential errors in data extraction. The data extracted will include general information about the article, and specific details about the instrument as well as the psychometrics. Findings will be extracted and assigned a level of credibility (Table 3).

### Assessment of methodological quality of included studies

We will apply the COSMIN checklist to evaluate the methodological rigor and results of the instruments [34]. The checklist consists in modules that enables specific criteria to be applied to certain tests. The flexibility of the checklist ensures that the same level of scrutiny is applied to evaluate various studies of instruments, even if they have conducted different psychometrics tests. Selected studies will be critically appraised by two independent reviewers for methodological quality using the COSMIN Scoring quality form boxes [48]. Any disagreements that arise between reviewers will be resolved through the evaluation by the third reviewer, who is a psychometrics expert (MS). The results of critical appraisal will be summarized in a table.

Following COSMIN guidelines, only the studies that have a “good” methodological quality in content validity will undergo data extraction analysis and synthesis. The quality of included studies will be considered in the analysis and will therefore be reflected in the findings and a grade will be given in conclusion of the systematic review [34]. If we have multiples studies for one PROM, all results per measurement property of a PROM will be synthesized. Then the results will be evaluated against the criteria for good measure proprieties to have an overall rating for the measurement property (Table 4) in order to give a grade.

**Table 4:** Rating the measurement properties of the PROM [35]

PROM	Study 1			Study 2		
	Rating	Rating	Rating	Rating	Rating	Rating
	+/−/ ?	+/−/?	+/−/?	+/−/?	+/−/?	+/−/?
	Rater 1	Rater 2	Consensus	Rater 1	Rater 2	Consensus
Structural validity						
Internal consistency						
Cross-cultural validity						
Measurement invariance						
Reliability						
Measurement error						
Criterion validity						
Construct validity						
Responsiveness						

### Evaluation of measurement proprieties for included instruments

The quality of each measurement property of an instrument will be evaluated using the quality criteria proposed by Terwee et al. [49] who participated in the group that developed the COSMIN checklist (see Table 4: Rating the measurement properties of the PROM).

Each measurement property will be given a rating result (‘+’ positive, ‘−’ negative, ‘?’ indeterminate, and ‘na’ no information available). There is currently no empirical method to pool together results of measurement properties; therefore, synthesis is recommended [50].

### Data synthesis

Research findings will be synthesize using the COSMIN “Scoring quality form boxes” tool [48]. These categories will then be subjected to a synthesis, in order to produce a single comprehensive set of synthesized findings that can be used as a basis for evidence-based practice. When pooling will not be possible, the findings will be presented in narrative form [51]. A narrative synthesis of instrument purpose, rigor, and findings will enable level of evidence recommendations for the selection of PROM.

### Best evidence synthesis: levels of evidence

As recommended by the COSMIN Group ‘a best evidence synthesis’ will be used to synthesize all the evidence on measurement properties of different instruments. The procedure used will follow the Grading of Recommendations, Assessment, Development, and Evaluation (GRADE) framework [34]. This procedure is a transparent approach to rate the quality of evidence that is often used in reviews of clinical trials. Under this procedure, the possible overall rating for a measurement property is ‘positive’, ‘negative’, ‘conflicting’ or ‘unknown’, accompanied by levels of evidence (‘strong’, ‘moderate’ or ‘limited’) (Table 5).

**Table 5:** Quality of evidence for measurement proprieties of the PROMs COSMIN scoring box tool ( [25, 35], p. 62)

	Overall rating	Quality of evidence	Overall rating	Quality of evidence	Overall rating	Quality of evidence
	+/−/?	High, moderate, low, very low	+/−/?	High, moderate, low, very low	+/−/?	High, moderate, low, very low
Content validity						
Relevance						
Comprehensiveness						
Comprehensibility						
Structural validity						
Internal consistency						
Cross-cultural validity						
Measurement invariance						
Reliability						
Measurement error						
Criterion validity						
Construct validity						
Responsiveness						

Three steps will be taken to obtain the overall rating for a measurement property. First, the methodological quality of a study on each measurement property will be done using the COSMIN checklist. According to COSMIN, measurement properties from inadequate quality studies may be included to contribute to ‘the best evidence synthesis’. Second, the quality of each measurement property of an instrument will be evaluated using Terwee’s quality criteria [37, 50]. Third, the rating results of measurement properties in different studies on the same instrument will be examined whether consistent or not. This best evidence synthesis will be performed by one author and then checked by a second author.

### Meta-bias(es)

Following COSMIN guidelines, the results from inadequate quality studies may be included when synthesizing or summarizing the results from all studies, or if the results of inadequate studies are consistent with the results of better quality [52].

## Discussion

This review will present a synthesis of eHL’s PROMs, their psychometric properties, and make recommendations for selection in practice and research. This review will provide some insight regarding the challenges of using self-report instruments in assessing eHL*.* We will systematically discuss the main issues in the testing of a subjective health measurement instrument in regard to existing systematic reviews in the eHL study domain (including the specification of the instrument characteristics: the chosen measurement paradigm, the context of use, the structure of the instrument and measurement proprieties). We will discuss operational issues related to the conduct of this systematic review of PROMs. The approach which consists of using eHealth resources has led to improvements in clinical outcomes and health service efficiency and might potentially improve access to care and reduce healthcare costs. For clinical practice, it is important to have a good instrument to appraise patients’ eHL level in order to correctly use eHealth resources and maximize benefits for the patient’s disease self-management. It is also essential to provide patients and providers with a rigorous evaluation of e-HL competencies that facilitate the use of eHealth in the relevant context.

## Abbreviations

eHL: eHealth literacy
eHealth: Electronic health
PROM: Patient-reported outcome measures
COSMIN: COnsensus-based Standards for the selection of health Measurement Instruments PRISMA Preferred reporting items for systematic reviews and meta-analysis
